# Summer Eastern North American Monarchs Do Not Use Magnetic Field for Directionality but Extreme Heat Alters Righting Behavior

**DOI:** 10.3390/insects17101052

**Published:** 2026-10-13

**Authors:** Samuel A. Shively-Moore, B. G. Chethana Chathuprabha, Patrick A. Guerra, Stephen F. Matter

**Affiliations:** 1 Department of Biological Sciences, University of Cincinnati, Cincinnati, OH 45221-0006, USA; mattersf@ucmail.uc.edu; 2 Department of Mathematical Sciences, University of Cincinnati, Cincinnati, OH 45221-0025, USA; belpagca@mail.uc.edu; 3 Department of Entomology and Plant Pathology, North Carolina State University, Raleigh, NC 27695, USA; paguerr2@ncsu.edu; 4 School of Environment and Sustainability, University of Cincinnati, OH 45221-0367, USA

**Keywords:** climate change, compass orientation, *Danaus plexippus*, migration, non-migratory, righting behavior

## Abstract

Summer Eastern North American monarch butterflies (*Danaus plexippus*) represent one leg of the annual multi-generational migratory-range expansion cycle of monarchs in North America, as they spread north and repopulate the breeding habitat. Migratory monarchs use compass orientation to fly southwest in the fall to their overwintering sites. Though summer monarchs do not have a specific migratory endpoint like fall monarchs, it is possible that summer monarchs migrate north using untested magnetic compass orientation mechanisms. We examined the role of magnetic cues in the righting orientation behavior of summer monarchs to test the hypothesis that summer monarchs are indeed migratory and display northward orientation. Response to magnetic cues can be obtained from a righting response assay, where orientation can be observed in the righting behavior of monarchs on a vertical plane. Because magnetic orientation can be affected by temperature, we hypothesized that if summer monarchs did not show a northward orientation, then exposure to high temperatures (with or without prior experience to high temperature) would induce a northward orientation. We found that summer monarchs would right themselves, but did not use a magnetic compass to orient northward. In addition, summer monarchs exposed to high temperatures did not right themselves consistently. Thus, our results reinforce that summer monarchs are non-migratory in the strict sense and do not use a magnetic compass for orientation, nor is magnetic orientation induced by high temperature; however, high temperatures can induce other behavioral changes. As average temperatures increase and extreme climate events are predicted to increase in frequency with climate change, it is important to identify the behavioral responses to these changes.

## Simple Summary

As a part of the annual multi-generational migratory-range expansion cycle of Eastern North American monarch butterflies (*Danaus plexippus*), the summer generations spread north and repopulate their breeding grounds, despite not having the same directional behaviors as the fall migratory generation. Though they may not have a specific migratory endpoint or use mechanisms to orient, they may use yet untested orientation mechanisms, such as a magnetic compass, to aid in their northward expansion and thus be actual migrants. If summer monarchs are migratory, we would expect migratory restlessness such as that observed in fall monarchs, who right themselves and orient in the expected direction, which is northward for summer monarchs. Because magnetic orientation can be affected by temperature, we hypothesize that if monarchs did not show northward orientation, then exposure to high temperatures consistent with summer thermal conditions would induce northward orientation. We found that summer monarchs did not use a magnetic compass to orient northward, with or without heat exposure. Furthermore, monarchs ceased to right themselves after long-term heat exposure. As average temperatures increase and extreme climate events are predicted to increase in frequency, it is important to identify the tolerance, buffering abilities, and thermoregulation mechanisms of butterflies.

## 1. Introduction

The behavior of the Eastern North American monarch butterfly (*Danaus plexippus*) serves as a remarkable example of a complex movement phenotype in response to a combination of environmental cues (e.g., photoperiod and temperature) and internal compass mechanisms during their long-distance annual fall migration from the northern United States and southern Canada to their overwintering habitat in central Mexico [1,2]. In spring, remigrants leave their overwintering habitat in Mexico and journey north to start repopulating the northern area of their range [3,4]. Spring remigrants produce the subsequent generation of springtime monarchs, which continue to expand north [3]. The offspring of springtime monarchs become the summer monarchs that further contribute to the northward range expansion throughout their breeding grounds of the northern United States and Canada east of the Rocky Mountains and complete the annual multi-generational migratory cycle. The generations of monarchs that disperse northward from the southern United States to reach and populate the summer breeding grounds have long been considered non-migratory, as they do not demonstrate the same repertoire of orientation specific behaviors compared to fall migrants (e.g., summer monarchs do not use a time-compensated sun compass for directionality) [1,3,4,5]. Migratory animals generally exhibit a syndrome, where they may express traits and behaviors in favor of movement and navigation toward a goal location or direction [4]. It is unclear what motivates the summer generations of monarchs to continue their northward expansion if they are not migratory, and what mechanisms they employ to facilitate northward expansion. As part of the annual multi-generational cycle, summer monarchs may use a different suite of cues or navigational mechanisms to move northward than in the fall [4]. Although they do not have a specific endpoint like fall monarchs, who target specific overwintering sites [3], it is possible that summer monarchs do migrate northward using a magnetic compass. For fall monarchs, a light-dependent magnetic compass serves as a backup orientation mechanism during migration to maintain southward (equatorward) flight on overcast days when directional sun cues are unavailable [6]. Here, fall monarchs use the inclination angle of the geomagnetic field as a directional cue to maintain southward flight during the fall migration [6].

There is increasing evidence that summer monarchs are capable of oriented movement in response to environmental cues [7,8]. Recent research has demonstrated that monarch butterflies, even when they are not fall migratory, can register multiple visual cues (phototaxis) to keep a directed course [7] and robustly orient in response to gravitational cues (negative gravitaxis) [8], suggesting that summer monarchs potentially use environmental orientation cues to direct movement in a similar way as fall and spring remigrant conspecifics. Summer monarch northward expansion, however, might instead be motivated by progressively increasing milkweed abundance (and limited by the range of milkweeds in northern latitudes), or the avoidance of heat stress as summer temperatures rise [4,5,9,10]. Despite ample research to understand what cues are important for fall migratory monarchs, it is not as well-known how summer individuals use environmental cues in their northward expansion.

As monarchs face constant external stimuli from their environment, determining how they interact with these sensory cues can help identify the important cues for their overall behavior [11,12]. Temperature can change a considerable amount in the short-term, and because of the small size and mass of butterflies, their body temperature can change dramatically as a result [13]. Butterflies must cope with extreme air temperature changes locally and employ multiple thermoregulatory strategies [13,14,15]. Temperature also has an important role in inducing orientation mechanisms for migrating monarchs. For example, cold temperatures during overwintering recalibrate the directionality of the Sun and magnetic compasses from south to north [16,17], thus hot temperatures, over a short or a long period of time, might induce and inform northward orientation for summer monarchs.

In addition to using temperature cues to calibrate compass mechanisms, butterflies may first respond more simply to changes in temperatures due to the low energy cost [15]. Wings can overheat quickly under high temperatures, so they may first adjust their basking posture to alter the position of their wings relative to the Sun or reduce their time basking [18], and conversely orient toward the Sun to warm during low temperatures. Monarch butterflies overwintering in Mexico will adjust their basking behavior to minimize sun exposure to their wings or they will glide above the colony to lose heat to the ambient temperature to avoid warming and using overwintering reserves [19]. Larger butterflies, like monarchs, are better able to regulate small fluctuations in body temperature and have high mobility, allowing them to access a greater range of microclimates, thus resulting in more behavioral thermoregulation [14,20]. Butterflies who behaviorally thermoregulate may not have evolved to have physiological attributes to regulate heat, and the risks and behavior associated with increasingly high temperatures may become more pronounced as global temperatures continue to rise [18,21,22,23,24].

**Figure A1:**
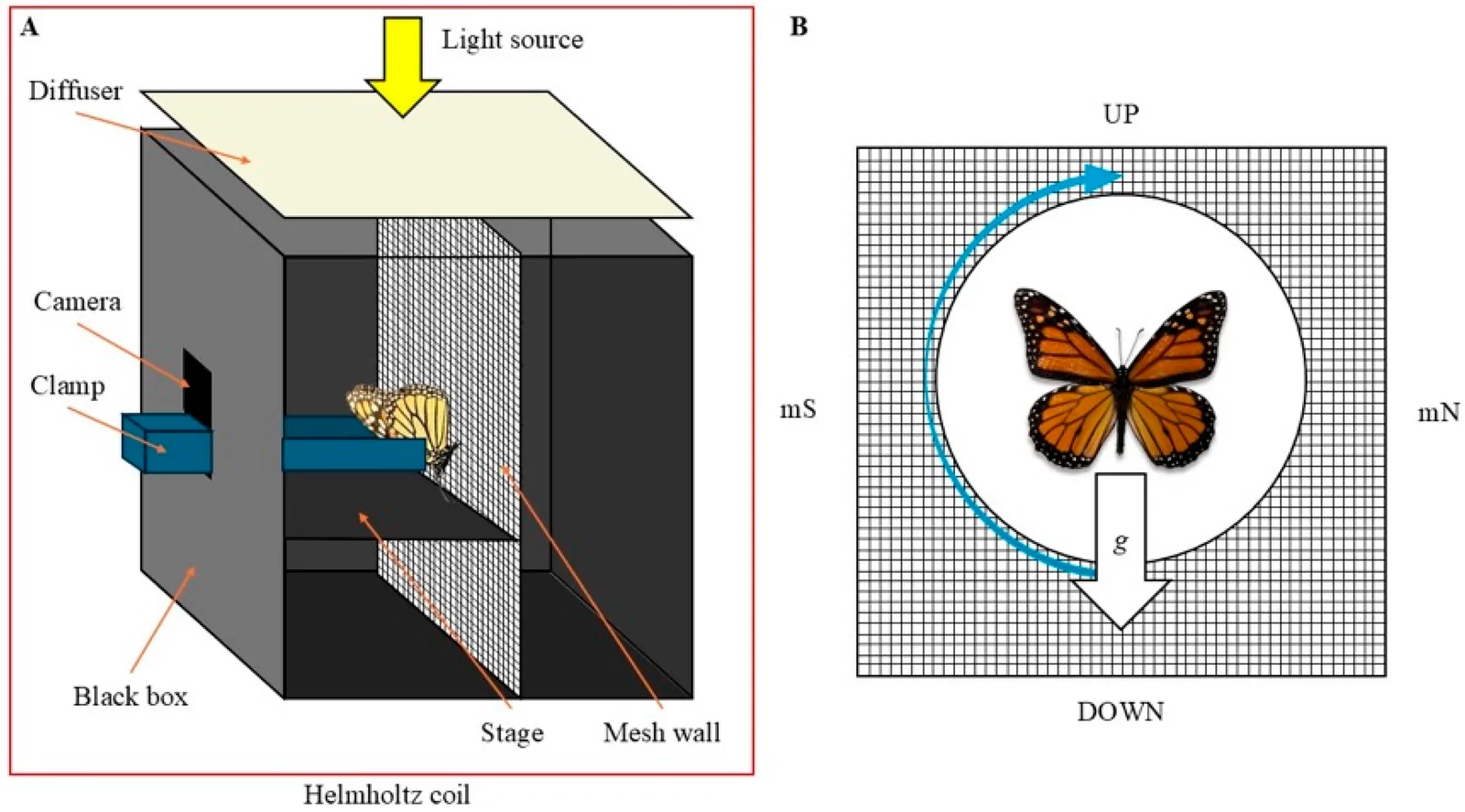
Righting response orientation assay construction. (**A**) Apparatus of righting response trial where monarchs are clamped head-down on a mesh wall and released to allow for free movement on the vertical plane. Camera and clamp were placed in the wall to view and control the monarchs movement prior to trial, and UV light was diffused from above. A stage sat directly below the clamp to rest on during the trial while the monarch had free movement. The black box containing the apparatus sat inside a Helmholtz coil system to control magnetic field information. (**B**) Diagram of orientation, where magnetic south (mS), magnetic north (mN), up and down are labeled. Monarchs start in the head-down position, and when released from the clamp, right themselves in response to gravity and orient in their desired direction. Blue arrow represents the hypothetical path of movement of monarch orientation. Gravity (*g*) is labeled to demonstrate the orientation of the mesh wall in space. Figure modified from Kendzel et al. [8].

Here, we examine the role of the magnetic compass in the orientation behavior of summer monarchs and test the hypothesis that they are migratory, since much of the sun compass work [5] was conducted prior to our understanding of the use of the magnetic compass by monarchs [6]. Like the temperature cues that change orientation direction in migratory monarchs [16], high temperatures may similarly induce orientation in summer monarchs. We predicted that summer monarchs use magnetic cues during their northward expansion. Furthermore, the ability to use the magnetic compass may be initiated by high temperatures. These hypotheses were tested using a righting response assay, in which a response to magnetic cues can be obtained by observing the orientation of the righting behavior of monarchs on a vertical plane (see section below; Figure A1) [8,17].

## 2. Materials and Methods

### 2.1. Animals

Both sexes of adult Eastern North American summer monarchs, *Danaus plexippus* (Linnaeus 1758) were collected on the wing from 24 June to 1 September 2025 at the University of Cincinnati’s Center for Field Studies (Harrison, OH, USA; 39.285° N, 84.742° W). All butterflies were first housed under control temperatures (see below) for a two-day period in a DigiTherm incubator (model: DT2-MP-47L, Tritech Research, Los Angeles, CA, USA), at 70% relative humidity, on a 15 h:9 h light:dark cycle. All monarchs were fed a 25% honey/water solution daily throughout the experiment.

### 2.2. Temperature Treatments

**Figure 1:**
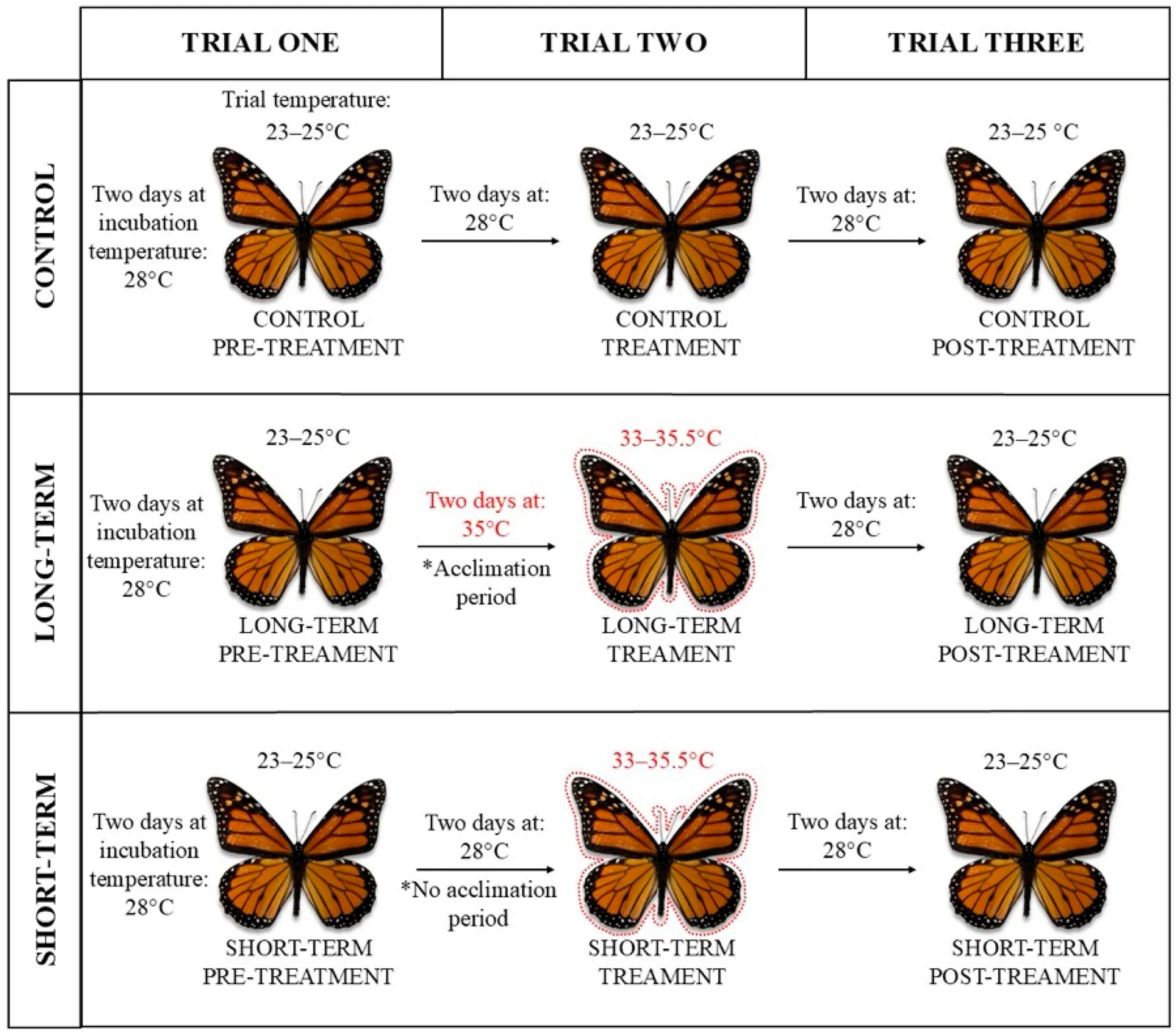
Temperature treatment regime for each trial and group. The control group was established as a baseline for summer monarchs throughout the trials to demonstrate their righting orientation behavior when there is no change to temperature. The long-term group was set up to examine the effect of high temperatures with acclimation on behavior and their behavior following a high temperature event. The short-term group was created to examine the effects of a temporary change in temperature (exposed to high temperature during trial only) and their behavior following a short high temperature event. Red dashed outline behind monarchs represents trials held at high temperatures, red text represents time spent in high temperatures, and *text indicates whether the monarchs were given an acclimation period.

To understand the effects of long- and short-term heat exposure on orientation behavior, we employed three thermal regimes. Summer monarchs were split into three groups differing in thermal exposure: a control group that did not experience high temperatures (control), a treatment group that was acclimated to high temperatures (long-term heat), and a group that was exposed to high temperatures but was not provided with an acclimation period (short-term heat) (Figure 1). All monarchs were first kept in the same incubator for two days at control temperature conditions: 28 °C during the light phase and 20 °C during the dark phase, based on the average 2024 summer temperature in Cincinnati, Ohio. Temperature data were collected from a meteorological station near the testing site (KOHCINCI680) from Weather Underground (https://www.wunderground.com/, accessed on 13 June 2025). Summer monarchs live between two to five weeks as adults [25]. Due to the unknown age of the captured monarchs and the initial low survivorship of individuals, the two-day period of acclimation was selected as a standard period after capture to increase the likelihood that a monarch would complete all trials without perishing. After the two days of acclimation to laboratory conditions, monarch orientation was tested in a Helmholtz coil using a righting orientation assay using local magnetic field information [8] (see next section) for the first trial to establish whether monarchs orient directionally northward using a magnetic compass, the expected directionality for summer monarchs if they are migratory.

Monarchs were then prepared for a second righting orientation trial to test their reaction to a change in temperature: monarchs in the control group and the short-term group continued to be held at the control temperature, but monarchs in the long-term group were transferred to a high temperature condition after the first trial. The high temperature was 35 °C for the light phase and 20 °C for the dark phase, based on the average high for summer 2024 in Cincinnati, Ohio collected from the same meteorological station as above (Figure 1). After two days, monarchs in the control group were tested in the righting assay at the control temperature, 28 °C, and monarchs in both the long-term and short-term groups were tested at 35 °C (Figure 1).

Monarchs in the long-term group were then returned to the control temperature incubator for two days, and the control and short-term monarchs continued to be held in the control temperature incubator. After two days, all monarchs were tested at the control temperature in the third righting assay (Figure 1).

Temperatures within the trial box were kept within a 3 °C range of the goal temperature: control temperatures within the box were between 25 and 28 °C and high temperature trials between 35 and 37.5 °C.

### 2.3. Magnetic Field and Trials

Kendzel et al. [8] and Shively-Moore et al. [17] demonstrated that monarch orientation and righting behavior (negative gravitaxis) occurred using a righting response assay. To test summer monarch orientation, we followed the same protocol as used in Shively-Moore et al. [17], where monarchs were clamped head-down and released to initiate a response to gravity where they right themselves and orient in response to the presented magnetic cues (Figure A1). If summer monarchs are migratory, we would expect a similar migratory restlessness as that observed in fall monarchs, who first right themselves upward and then orient in the expected southward (equatorward) direction [8,17], but we would expect summer monarchs to instead orient northward (poleward) (similar to *Zugunruhe* [26]).

In the righting response orientation trials, butterflies were placed inside a Helmholtz coil system that allowed us to control the magnetic field that the butterflies would experience during the trials. The coils were used to keep the magnetic field consistent with that of the surrounding area and aligned with the local geomagnetic conditions (Cincinnati, OH, USA; 39.134° N, 84.517° W). Trials were completed in a darkroom with the righting apparatus in a black box (LWH: 32 cm × 20 cm × 34 cm) inside the coil system. Centered inside the black box was a mesh wall (32 cm × 32 cm), perpendicular to the ground to serve as the plane of movement for the monarchs during the trials. At the top of the box was a source of diffused, non-directional light that contained the relevant UV light stimulus that elicits magnetic orientation in monarchs [6,8,17]. In the wall behind the monarch, a plastic clamp and a camera (model: HDR-CX440, manufacture: Sony Corporation, Tokyo, Japan) were embedded to control and record the monarch’s movement. Below the clamp between the mesh wall and the black box was a stage to rest the clamp during the trial within the box below the monarch.

In all righting behavior trials, monarchs were held head-down before releasing and allowing free movement on the vertical plane. Orientation scores were noted once the monarch in a trial maintained a position for at least 30 s. The final orientation of a monarch during a trial was measured with a protractor from the video recording.

While observing their righting orientation behavior, we found that some monarchs did not stay on the vertical plane (jumping, dropping, or flying off the testing apparatus) under all experimental conditions. Dissipating high temperatures by flight has been observed in monarchs [18,19], and movement away from heat is consistent with heat avoidance found in monarchs and other animals [15,27,28]. If a monarch did not stay on the vertical plane for a maximum of three attempts, that trial was recorded as “failure to right” and used to analyze alternative righting behavior, particularly in response to high temperatures.

We compared the righting behavior of each group of butterflies to compare high temperature treatments (long-term, short-term, and control), and how this alternate behavior compared to the control group in a repeated measures design (pre-treatment, treatment, and post-treatment; Figure 1).

### 2.4. Data Analysis

We first assessed directionality using Rayleigh’s test to determine whether groups had a mean orientation in each treatment and trial and were thus suitable for further analysis. A V-test was then used to determine whether the mean orientation of each treatment group was different than the expected null orientation of 0 degrees (response to gravity with no orientation in response to the magnetic field). Only two individuals in both the long-term and short-term groups completed the righting behavior across all three trials, therefore a Bayesian circular mixed-effects model was used to determine whether the individuals’ mean orientation differed across trials and among treatment groups [29]. We performed post-hoc-like analyses comparing parameter estimates and their upper and lower bounds. Summary statistics and comparisons between treatments were calculated in R version 4.3.1 [29] and visualized in the program Oriana version 4 (https://www.kovcomp.co.uk/oriana/, accessed on 23 October 2025); Kovach Computing Services, Pentraeth, Isle of Anglesey, UK).

Fisher’s exact tests were used to determine whether there was a significant relationship between the temperature condition of the trial and the behavior (normal versus alternate). For significant results, we used post hoc analyses with Simes correction [30].

## 3. Results

### 3.1. Lack of Evidence of Summer Monarchs Using Magnetic Cues for Orientation

**Figure 2:**
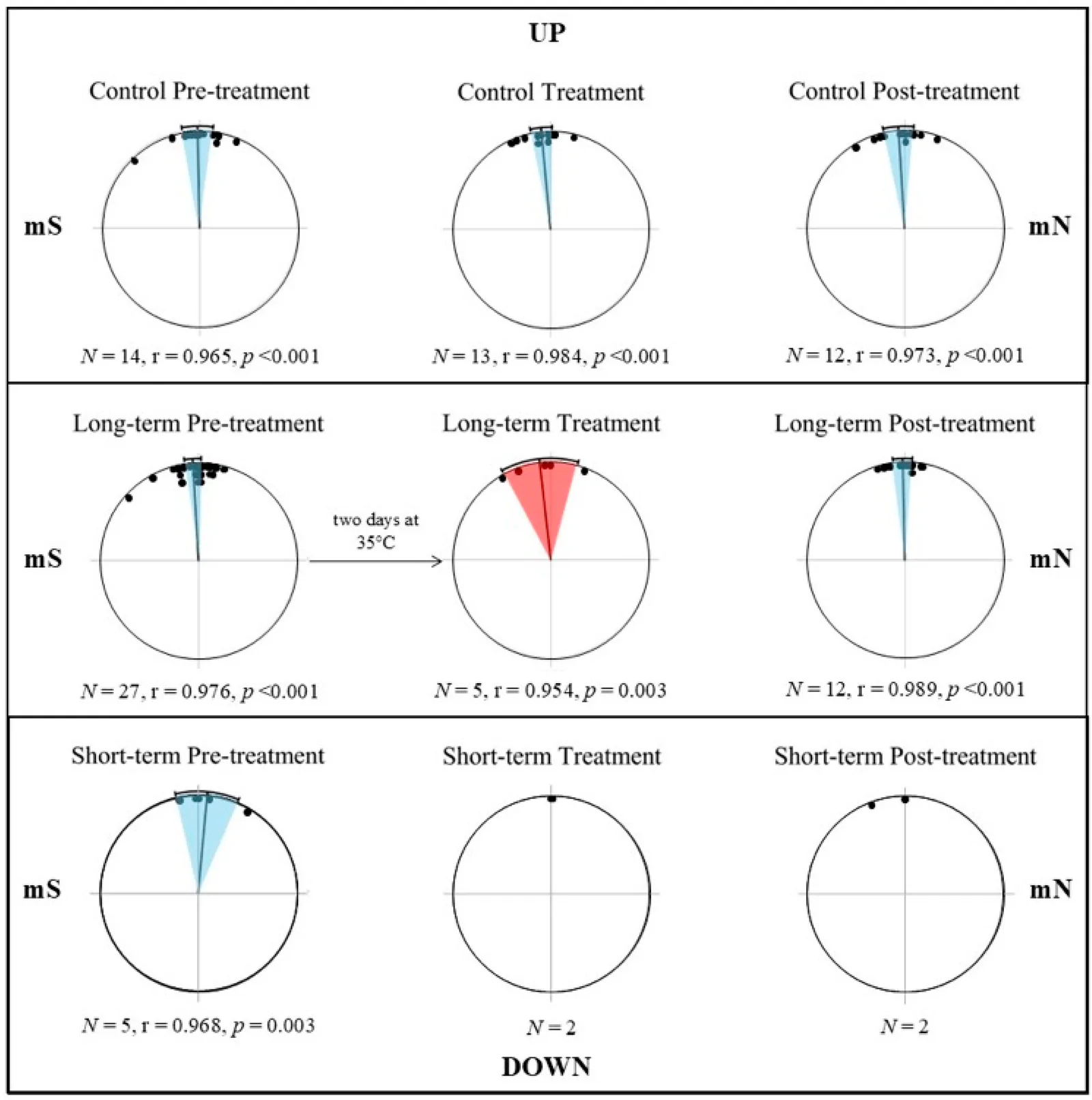
Righting orientation direction did not differ regardless of treatment group or trial. Monarchs were tested in three distinct treatment groups: (1) control, which received no change to temperature during housing or trials, (2) long-term, which received a period of high temperature and a high temperature trial, and (3) short-term, which received only a high temperature trial with no change to housing temperatures. Summary statistics below the circle diagrams show the number of individuals tested (*N*), the length of the mean vector (r), and the Rayleigh’s test significance (*p*). For each diagram, each dot indicates the heading of an individual butterfly, the arrow indicates the mean group orientation, shaded area is the confidence interval (red = high temperature trial, blue= average temperature trial), and mN is magnetic north, and mS is magnetic south.

Throughout our study, all groups that performed righting behavior displayed negative gravitaxis and righted themselves into the expected head-up vertical position (there was a significant mean group orientation across all trials, with mean orientations not different than zero; Table 1 and Figure 2).

We then addressed whether all monarchs oriented similarly. There was no difference in individual behavior based on treatment or group; all coefficients overlapped with zero (Table 2). Monarchs performed negative gravitaxis but showed no additional northward orientation using gravity cues regardless of their treatment, indicating that summer monarchs do not use magnetic cues for orientation, nor does temperature induce a magnetic response.

### 3.2. Summer Monarchs Demonstrated a Response Consistent with Thermoregulation

**Figure 3:**
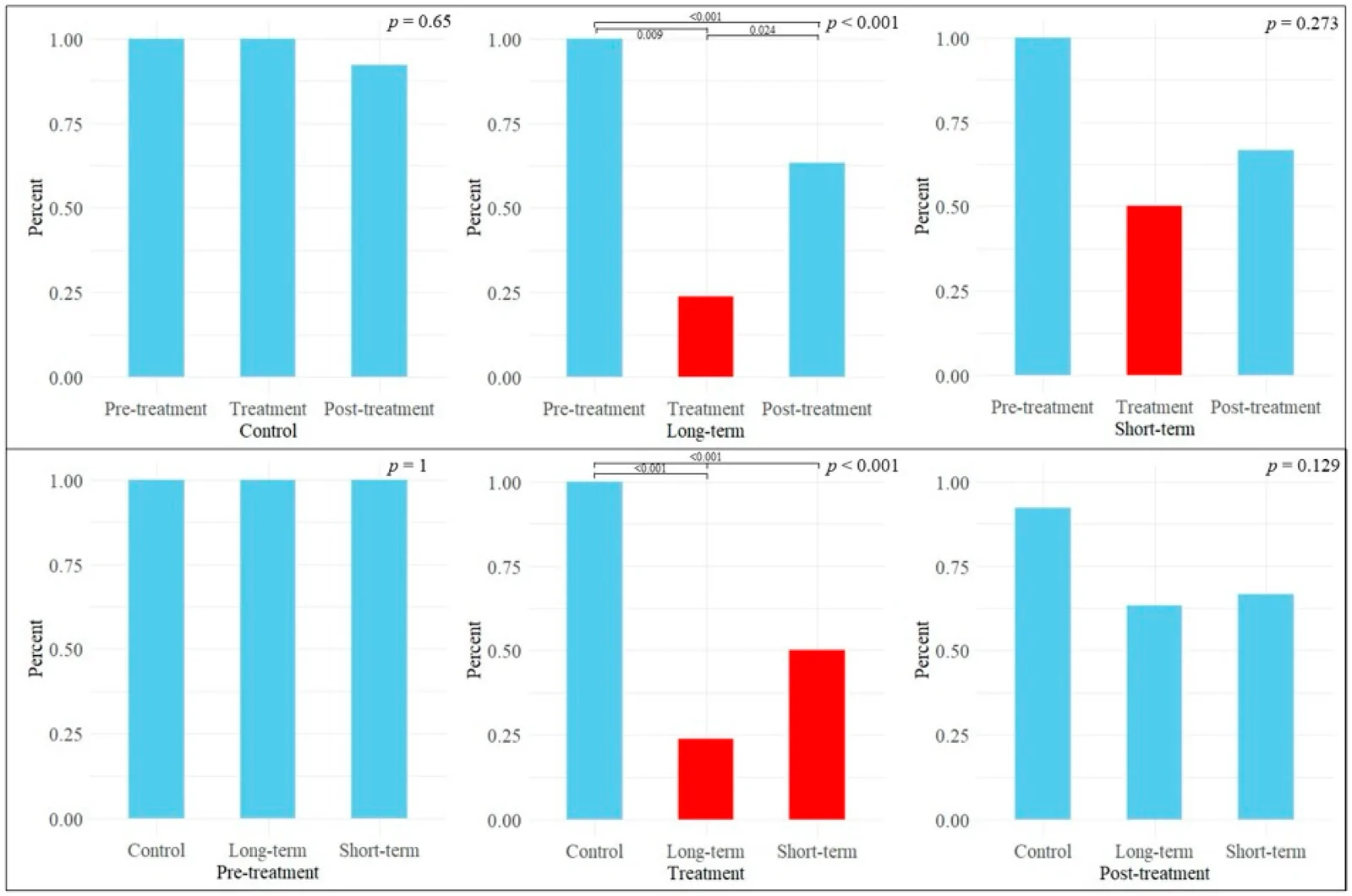
Exposure to a long-term period of high temperatures caused reduced frequency in completion of the righting orientation trials. Monarch behavior was compared across each treatment group (**top** row: control, long-term, and short-term) across all trials and how it appeared in each trial (**bottom** row: pre-treatment, treatment, post-treatment) across all groups. Statistics show the Fisher’s exact *p*-value (*p*) for each graph, and Simes correction *p*-values for each significant comparison (red = high temperature trial, blue = average temperature trial).

High temperatures altered the righting behavior of summer monarchs, wherein monarchs were less likely to complete the righting behavior once exposed to long-term heat. Pre-treatment, when all monarchs were exposed to the control temperature only, groups consistently displayed negative gravitaxis, as all monarchs righted themselves (first trial across all groups—control, long-term, and short-term; Table 3; Figure 3). After treatment (second trial), monarchs not exposed to high temperatures completed the righting trials, but a portion of those in the long-term group failed to right themselves, leading to significant differences between treatment groups (Table 3). Control group monarchs had no change in righting behavior across trials. Butterflies in the long-term treatment group only partially recovered post-treatment, as some recovery is indicated by the difference between treatment and post-treatment (Table 3, *p* = 0.024), but only partial by a significant difference between pre- and post-treatment (Table 3, *p* = 0.009). Fewer monarchs righted themselves in the treatment and post-treatment trials after short-term heat exposure; although this was not significant in the comparison of pre vs. treatment vs. post (Table 3, *p* = 0.273).

Summer monarchs that had no long-term exposure to high temperatures did not have any difference in righting behavior. In contrast, monarchs had altered righting behavior once long-term exposure to high temperature was introduced; even after they were returned to control temperatures, they did not right themselves consistently. All summer monarchs in the long-term group completed the pre-treatment control conditions righting trial; however, they did not right themselves across all temperature trials. Specifically, less than a quarter of the long-term group completed the trials in the high temperature treatment condition (23.81%), and just under two-thirds completed the control temperature post-treatment condition (63.16%) (Table 3; Figure 3), indicating a severe detrimental effect followed by partial recovery. Similarly, all summer monarchs in the short-term group completed the pre-treatment righting trial, but only a portion completed the high temperature treatment (50%) and post-treatment (66.67%) trials (Table 3, *p*= 0.273). We found no difference in the righting assay completion of monarchs in the control group (no temperature change throughout) across all trials (pre-treatment = 100%, treatment = 100%, post-treatment = 92.31%) (Table 3).

## 4. Discussion

The results of our study provide further evidence that summer Eastern North American monarchs are non-migratory in that they do not use a magnetic compass or sun compass to orient northward during their summer range expansion in eastern North America. It has been reported that summer monarchs do not exhibit directional or group-oriented flight based on tested cues [5,31,32]. Summer monarchs righting themselves to a head-up vertical position without additional orientation (an orientation of zero degrees) would indicate that they were not directionally orienting in response to magnetic cues, but rather responding to gravity alone [8]. These results demonstrate that summer monarchs only righted themselves but did not orient relative to the magnetic field, as observed in migratory fall monarchs [8,17] or experimentally primed spring remigrants [17].

We hypothesized that summer monarchs may use magnetic cues for northward flight to escape increasingly hot conditions as summer progresses; however, we found no support for this hypothesis in our results. However, monarchs may use other cues that orient their flight northward. Visual cues primarily guide the fall migratory response [8], and it is possible that summer monarchs use visual cues to maintain a direction when they are not migrating [7]. Since we used diffused light, we did not test the possibility that they may use directional light cues for orientation in response to changes in temperature [5]. Additionally, northward expansion may be driven by the increasing abundance of milkweed as summer progresses, but equally limited since milkweed does not grow in latitudes above their breeding grounds in southern Canada [33].

Environmental cues, like the magnetic field, initiate a physiological mechanism in migratory animals (e.g., sea turtles [34], spiny lobsters [35], pigeons [36]) that they respond to directionally throughout their migration. Monarch butterflies utilize magnetic cues to orient southward to their overwintering sites and northward in primed remigrants [6,8,17], but there is less known about their multi-generational northward expansion. Multi-generational long-distance migration has been observed in other butterfly species, but their orientation and navigational mechanisms are not as clear or robust as fall migratory monarchs [37].

For some butterfly species, oriented geographic expansion may in part be due to seasonal shifts, such as northward in the spring in the Northern Hemisphere [38]. The summer generation of monarchs are more aligned with this pattern, breeding continuously throughout their northward range expansion. For example, from the Nymphalidae family, the painted lady (*Vanessa cardui*) are known for their extreme multigenerational migratory cycles across North America, Africa, Europe, and Asia [39]. The behavior of *Vanessa* is not dissimilar to that of summer monarch butterflies. During their migration, the painted lady continuously reproduce throughout their range, and like summer monarchs, are as equally fecund as non-migratory butterfly species [40], but migratory monarchs uniquely enter diapause for their southward migration and overwintering [41]. There is a strong association between winds from North Africa and the arrival of the painted lady in northeastern Spain [42], providing some evidence that wind may aid their migration, but currently, neither the painted lady or the summer monarch have a clear mechanism for oriented movement during range expansion [38,43]. Temperature, photoperiod, prevailing wind, population density, and host plant senescence may serve as additional motivation for the summer range expansion of butterfly species, without the use of compass orientation or specific map sense [37,42]. Considering the similarity in strategy for range expansion across hundreds of butterfly species and the importance of the aforementioned environmental cues, monarchs may share the same ancestral state as other butterflies. However, since they uniquely differ in behavior for their fall migration, it may be the case that the migratory syndrome of fall migratory monarchs is a novel evolutionary state.

Other motivations may in the short-term change behavior that ultimately leads to longer distance movement. Butterflies bask to warm their wings, and as temperatures increase, the time they spend basking decreases to avoid overheating [18]. In natural conditions, butterflies can position their wings to reduce surface area or reflect more sunlight [13], or they may change location to a shady area to reduce their temperature [27,28]. Some butterflies have temperature-induced plasticity (e.g., heat shock proteins), which can expand their thermal tolerance for a time, but are energetically costly to maintain, thus limiting exposure to high temperatures would be energetically less costly [44,45]. In our results, introducing long-term exposure to high temperatures reduced the number of instances where they righted themselves, while short-term exposure to high temperatures had no effect on the behavior of summer monarchs when compared to the control group. Those in the long-term high temperature treatment may already have had a high core temperature to start and therefore avoided longer exposure more urgently than ones that had short-term exposure, with no apparent acclimation to the high temperatures [13]. We did not specifically provide shade in the righting assay box; however, in some instances, monarchs would drop under the stage where the diffused light was not available rather than complete the righting assay (n = 27).

Long-term exposure to high temperatures before a trial affected the outcome of the behavior; treatments with no high temperature and no long-term exposure showed no significant difference among trials. Gravity serves as an important cue for monarch orientation and holds greater importance than their primary orientation cue (light) [8]. A cue strong enough to forego a response to gravity, or to lose the ability to respond to gravity, indicates the importance of overheating in their overall behavior [11]. Butterflies avoid risks of overheating by using multiple thermoregulatory strategies including seeking refuge from high temperature conditions [13,14,15]. Therefore, monarchs jumping off the test wall or that flew off to land at the bottom of the apparatus during the trials might be displaying positive gravitaxis, and under natural conditions, generally northward movement if they are moving away from sunlight. While some summer monarchs actively jumped or flew off the screen, it is possible that the few that dropped may not have been able to hold on as the heat compromised their behavior due to increasing energetic costs [44]. If the improper orientation was due to a behavioral response, it may prove to additionally serve monarchs multi-generationally and drive their migration northward as summer temperatures increase [9,22,23], but could be seriously threatening if high temperatures weaken monarchs into immobility [10,46].

## 5. Conclusions

The results of this study reaffirm that summer monarchs are not migratory. In addition to not using a time-compensated sun compass, monarchs in this study did not show magnetic orientation, as would be expected if they are migratory and are migrating northward. There was, however, a change in behavior in response to high temperatures, that of ceasing to right themselves after long-term heat exposure. Summer monarchs demonstrated that prolonged high temperature was more important than acute heat exposure, and that they can at least partially recover from long-term exposure to high temperatures after experiencing a period of cooler, average temperatures. Animals can modify their experience of their environment through behavior; butterflies adjust their body posture or seek local microhabitats to avoid high temperatures [18,47]. As average temperatures increase and extreme climate events are predicted to increase in frequency with climate change, it is important to identify the tolerance, buffering abilities, and thermoregulation mechanisms of butterflies to these long-term changes [14,27]. Although summer monarchs do not use a magnetic compass to orient in later summer generations, the role of other cues is still unknown in their northward range expansion.

## Figures and Tables

**Table 1:** Summary of statistical results for the group orientation of summer monarchs organized by treatment group (control, long-term, or short-term) and the trial (pre-treatment, treatment, and post-treatment). For each trial, we aimed for sample sizes of 14 for the control, 27 for the long-term, and 5 for the short-term, but not all individuals completed the trial presented. *N* = sample size, µ = mean direction, Rayleigh Z= critical value, r = magnitude of mean vector, u = critical value, and *p* = *p*-value.

Treatment Group	Trial	Trial	*N*	µ	Rayleigh Test			V-Test	
		Temperature (°C)			Z	r	*p*	u	*p*
Control	Pre-treatment	21–23	14	359°	13.043	0.965	<0.001	5.106	<0.001
	Treatment	21–23	13	355°	12.598	0.984	<0.001	4.998	<0.001
	Post-treatment	21–23	12	357°	11.361	0.973	<0.001	4.758	<0.001
Long-term	Pre-treatment	21–23	27	357°	25.71	0.976	<0.001	7.159	<0.001
	Treatment	33–35.5	5	354°	4.547	0.954	0.003	2.997	0.001
	Post-treatment	21–23	12	359°	11.735	0.989	<0.001	4.843	<0.001
Short-term	Pre-treatment	21–23	5	5°	4.69	0.968	0.003	3.049	0.001
	Treatment	33–35.5	2	-	-	-	-	-	-
	Post-treatment	21–23	2	-	-	-	-	-	-

**Table 2:** Summary of Bayesian circular mixed-effects model fixed effects results. µ = mean difference, LB = lower bound highest probability credible interval, and UB = upper bound highest probability credible interval. The Bayesian analysis converts angles into two-dimensions by taking the cosine (Component I) and sine (Component II) to remove circularity. If the difference in parameter estimates of either or both components do not overlap zero, it indicates a significant difference between groups.

Linear Coefficients	Comparison	µ	LB	UB
Component I	Control vs. Long-term heat	0.686	−2.468	4.001
	Control vs. Short-term heat	2.946	−1.237	7.510
	Long-term vs. Short-term heat	2.162	−2.816	7.565
	Pre-treatment vs. Treatment	−0.447	−3.192	2.213
	Pre-treatment vs. Post-treatment	1.169	−1.461	3.572
	Treatment vs. Post-treatment	0.810	−1.616	3.370
Component II	Control vs. Long-term heat	0.657	−0.252	1.546
	Control vs. Short-term heat	−0.484	−1.468	0.465
	Long-term vs. Short-term heat	−1.135	−2.329	0.100
	Pre-treatment vs. Treatment	−0.121	−0.817	0.594
	Pre-treatment vs. Post-treatment	0.200	−0.525	0.949
	Treatment vs. Post-treatment	0.076	−0.638	0.808

**Table 3:** Summary of Fisher’s exact test results of summer monarch behavior with Simes correction organized by treatment group and trial comparison. * indicated significance, *N* = total and *p* = *p*-value. Simes correction *p*-value is significant if it is below threshold value.

		Fisher’s Exact		Simes Correction			
Behavior Trial	Comparison	*N*	*p*	Comparison	n	Threshold	*p*
Pre-Treatment	Control vs. Long vs. Short	46	1.00	-	-	-	-
Treatment	Control vs. Long vs. Short	38	<0.001 *	Control vs. Long	34	0.0125	<0.001 *
				Long & Short vs. Control	38	0.0250	<0.001 *
				Control vs. Short	17	0.0375	0.044
				Long vs. Short	25	0.05	0.549
Post-treatment	Control vs. Long vs. Short	35	0.129	-	-	-	--
Control	Pre vs. Treatment vs. Post	40	0.650	-	-	-	-
Long-term	Pre vs. Treatment vs. Post	67	<0.001 *	Pre vs. Treatment	48	0.017	<0.001 *
				Pre vs. Post	46	0.033	0.009 *
				Treatment vs. Post	40	0.05	0.024 *
Short-term	Pre vs. Treatment vs. Post	12	0.273	-	-	-	-

## Data Availability

The data used in this study are publicly available and can be found at the University of Cincinnati’s Scholar@UC digital repository (https://doi.org/10.7945/5edh-4e17, accessed on 8 August 2026), made available under the Open Data Commons Attribution License: http://opendatacommons.org/licenses/by/1.0/ (accessed on 8 August 2026).

## Appendix A

Summer monarch orientation was tested using the same protocol as used in Shively-Moore et al. [17], in a righting response orientation assay nested in a Helmholtz coil system.
